# Staying connected: implementing avatar robots at schools in Germany and Japan

**DOI:** 10.3389/fdgth.2024.1273415

**Published:** 2024-06-05

**Authors:** Celia Spoden, Arisa Ema

**Affiliations:** ^1^ German Institute for Japanese Studies, Tokyo, Japan; ^2^ Tokyo College, The University of Tokyo, Tokyo, Japan

**Keywords:** school absenteeism, childhood illness, children with disabilities, telepresence robots, avatar robots, Japan, Germany

## Abstract

**Introduction:**

With advancements in communication technologies and internet connectivity, avatar robots for children who cannot attend school in person due to illness or disabilities have become more widespread. Introducing these technologies to the classroom aims to offer possibilities of social and educational inclusion. While implementation is still at an experimental level, several of these avatars have already been introduced as a marketable service. However, various obstacles impede widespread acceptance.

**Methods:**

In our explorative qualitative case study we conducted semi-structured interviews with eight individuals involved in the implementation of the avatar robots AV1 in Germany and eleven participants involved with implementing OriHime in Japan. We analyzed and compared implementation processes, application areas, access and eligibility, and the potential and limitations of avatars at schools.

**Results:**

We identified structural similarities and differences in both countries. In the German cases the target is defined as temporary use for children who cannot attend school in person because of childhood illness, with the clear goal of returning to school. Whereas in Japan OriHime is also implemented for children with physical or developmental disabilities, or who cannot attend school in person for other reasons.

**Discussion:**

Our study suggests that avatar technologies bear high potential for children to stay socially and educationally connected. Yet, structures need establishing that grant equal access to avatar technologies. These include educational board regulations, budgets for funding avatar technologies and making them accessible to the public, and privacy protection standards that are adequate, yet do not create implementation hurdles that are too high. Furthermore, guidelines or training sessions on technical, educational and psychosocial aspects of including avatar technologies in the classroom for teachers are important for successful implementation. Since our Japanese cases suggest that expanding the area of application beyond childhood illness is promising, further research on the benefits for different groups is needed.

## 1 Introduction: school absenteeism and avatar robots at schools

School absenteeism is said to lead to educational and social setbacks (1, 2) and may cause students to feel isolated and experience loneliness and depression (3). Several conditions can lead to school absenteeism, with different absence patterns in education. Furthermore, many children experience psychosocial or school-related issues on return to school (1, 2, 4).

The conventional way to secure the education of students with childhood illness or injuries are hospital schools, home tuition or sending schoolwork to the child (5). Yet, these services do not necessarily support students' social needs. The connection to their school community is easily lost (6) which makes returning to school more challenging (2). One way to support the educational and social needs of students and avoid social isolation is to introduce information technology-based communication tools into schools. Although there were first pilot studies on introducing telepresence robots for children in school settings as early as 1997 (7), it was not until advancements in communication technologies and internet connectivity that their use became more widespread (5).

Especially since the mid-2010s, there have been pilot studies on prototypes and the implementation of telepresence robots or avatar technologies in schools (3, 4, 6). There are mobile videoconferencing technologies showing the student controlling the telepresence robot on a monitor (3, 5–9) and stationary systems that do not transmit the video of the student (1, 2, 10–15). First studies have explored expectations of students and teachers (2), student and teacher experiences, and the potential and challenges for social inclusion (3, 4, 16). Studies have also outlined what telepresence robots mean for teachers and administrators in terms of training (1) and support, and suggested drafting guidelines for handling telepresence robots in the school environment (6).

The current trend is to introduce telepresence or avatar technologies in educational settings as a means for attending classes and interacting with peers. Implementation remains at an experimental level, yet several of these devices have been introduced as marketable services with numerous schools presented with implementation opportunities. However, they face various obstacles which hinder the adoption of these new technologies. Therefore, it is crucial to examine the experiences during the initial stages of implementation to determine the potential and limitations of these technologies, what hinders equal access to them, and what technical, financial, educational, social and psychological support is needed.

We conducted an explorative qualitative study on avatar technologies at schools in Germany and Japan and chose a cross-national multiple case study design (17). This approach offered an opportunity to sensitize the researchers for sociocultural particularities through comparison (for the choice of countries see the methods section). Since in both countries the introduction of avatar robots into the classroom is a recent phenomenon, the process of implementation had a prominent role in our explorations and interviews with study participants.

Our research questions were: (1) Which participants in each country are involved in setting-up avatar projects in schools and what challenges do they face? (2) What are the areas of application and who is eligible for participation in an avatar school project? And (3) What potential and limitations of avatar technologies do our research participants identify?

Our findings compare both countries, highlighting common issues and different approaches. The discussion contextualizes our findings with the literature on telepresence robots and avatar technologies. In the conclusion, we point out areas for further research. Although we also conducted interviews with students operating avatar technologies and their parents, in this article we focus on the interviews with study participants involved in the process of implementing avatars. We will present our findings on the experiences of students and their parents elsewhere.

## 2 Methods

**Figure 1:**
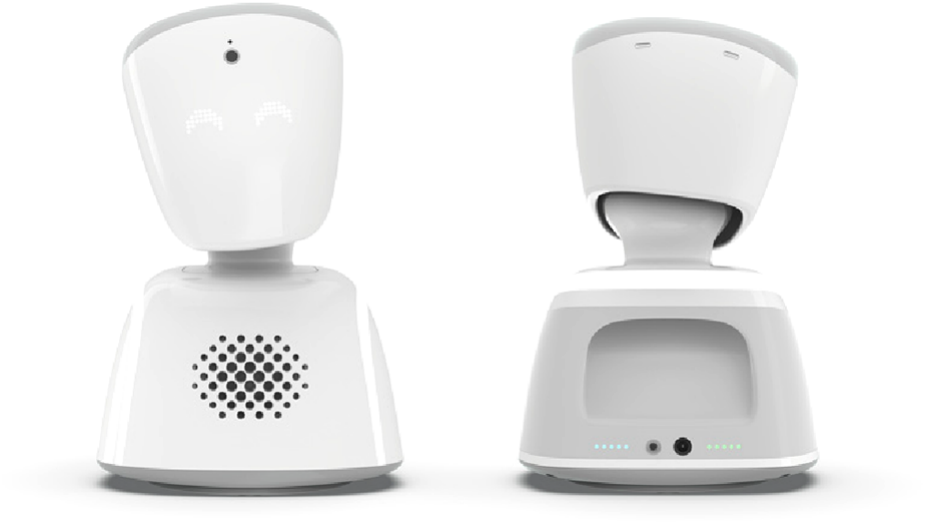
The avatar robot AV1 © No Isolation.

**Figure 2:**
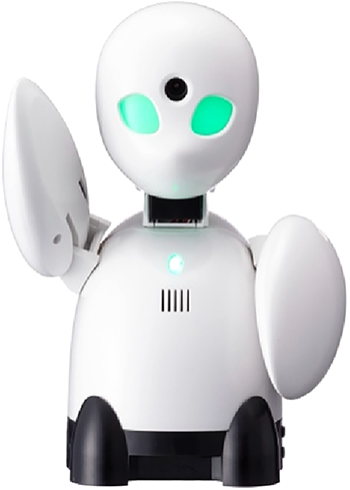
The avatar robot OriHime © OryLab Inc.

In this section, we first describe the study design. Then we introduce the avatar robots AV1 (Figure 1) and OriHime (Figure 2). We then describe the recruitment process, including detailed information about our study participants. Finally, we explain our data collection methods and analysis. For presenting our methods, we follow the consolidated criteria for reporting qualitative research (COREQ) (18).

### 2.1 Study design

The first author of this article is a senior research fellow at a German research institution in Japan, with a background in Japanese Studies and Philosophy (Dr. phil). The second author is an associate professor at a Japanese university and trained in Science and Technology Studies (PhD). Both authors reside in Japan, work together on the avatar robot OriHime from OryLab Inc., and are trained in qualitative methods.

Since the introduction of avatar technologies in schools is a recent phenomenon, we decided to conduct an explorative, qualitative case study. The interview data discussed in this article is part of a broader, ongoing study on the opportunities and risks of using avatar technologies in schools, approved by the ethics board from The University of Tokyo (No. 22–422).

As we wanted to sensitize ourselves to the sociocultural particularities, we choose a cross-national multiple cases design (17). The country of comparison was decided for practical reasons as one of the authors works for a German research institution in Japan and is regularly in Germany. Furthermore, the introduction of avatar robots in German and Japanese schools had started around the same time, so the duration of avatar project experience would likely be similar.

To the best of our knowledge the avatar robot OriHime is mainly used in Japan and has only occasionally been utilized in Denmark (2). We searched for a comparable device and AV1 from the Norwegian company No Isolation seemed promising.

### 2.2 The avatar robots AV1 and OriHime

Our study compared two avatar robots, AV1 (Figure 1) in Germany and OriHime (Figure 2) in Japan. AV1 was developed by the Norwegian start-up No Isolation in 2015 and its prototype implementation was in August 2016 (4). The aim of No Isolation is “to reduce loneliness and social isolation by developing warm technology and knowledge” (19). AV1 is advertised by the slogan “The child's eyes, ears and voice in the classroom” (20).

OriHime was developed by Yoshifuji Kentarō in 2010. He had been absent from school from ages 11-14 due to an illness and experienced this time as incredibly lonely. That became his motivation to develop technology that connects people. He founded his OryLab Inc. in 2012 and made “eliminating human loneliness” (12, 21, 22) his mission.

Table 1 shows the similarities and differences between the two avatar robots. Both types are designed to be placed on a classroom desk as a proxy and have heads that can turn and allow the student to look around with a camera. One important feature is that they only transmit video from the classroom to protect the student's privacy. They are equipped with speakers and a microphone. While OriHime expresses emotions through gestures with its wing-like arms, AV1 does so with its LED eyes. Both have a simple on/off button. In the standby modus they look like they are asleep. When someone logs in, the robots lift their heads and the “eye lights” turn on. This looks as if the robots are “awakening” and signals that someone is present and operating the technology. Both robot types are remotely controlled via a tablet, smartphone or computer. Additionally, AV1 has special functions designed for the classroom: a blinking head for “raising the hand,” a blue head signaling that the student does not feel well and only wants to listen, and three different volume levels. Important differences in privacy are that several people can have access to OriHime simultaneously. In contrast, the login to AV1 is password protected and limited to one person for the usage period. Furthermore, recording videos and taking pictures is impossible and prohibited with AV1, whereas OriHime can take pictures.

**Table 1:** Comparison of the avatar robots OriHime (OryLab Inc.) and AV1 (No Isolation).

	OriHime	AV1
**Operation**	Robot: simple on/off button; intuitive user app on mobile device	
**Video transmission**	Only video of the classroom is transmitted, no video of the user	
**Features**	Camera, microphone, speaker	
**Mobility**	Stationary, head moveable for looking around	
**Options for non-verbal communication**	**Arms/wings** for gestures and to express emotions	LED **eyes** to express emotions
**Functions**	Lifting an arm/wing for “raising the hand” Gaze control	White blinking of the head to “raise the hand” Blue glowing head to signal one only wants to listen Three different volume levels
**Login and password protection**	**Several people** can log in from their device simultaneously (optional)	**Login limited to one person**, password protection for the usage period
**Recording options**	Video recording **not possible**; taking pictures and screenshots **possible **	Video recording, taking pictures and screenshots **not possible**

### 2.3 Recruitment

Recruitment for the study was pursued through multiple routes. Initially we conducted a media search for telepresence robots in both countries and identified main stakeholders. Since the initiators and structures differed in the German and the Japanese cases, this was also reflected in the recruiting process.

#### 2.3.1 Recruitment and study participants in Germany

The first author contacted AV1 school projects and No Isolation in Germany via email. Following an initial interview, coordinator 1 of an avatar school project in a hospital introduced us to possible study participants from schools. No Isolation Germany also participated and informed possible study participants. A second avatar school project coordinator from another hospital was introduced to us via this connection.

The interviews in Germany took place from August until December 2022. Besides the two coordinators and No Isolation representative, we interviewed teachers, students, and with elementary school students, also their parents. Originally, we had planned to do participant observation in the classroom. However, since students with longtime childhood illness are especially vulnerable, the coordinators and No Isolation chose to introduce us only to cases where the student had recovered and returned to school.

In total we interviewed 15 individuals. For the research questions of this article, we selected the interviews with No Isolation Germany, two avatar school project coordinators based at hospitals, and interviews with teachers who had experiences introducing an avatar in class. Therefore, we based the analysis of this article on interviews with eight individuals (Table 2).

**Table 2:** Interview participants Germany: information on affiliation, recruitment route and interview setting.

No	Affiliation	Role	Recruitment route	Date interviewed
1	Hospital 1	Avatar school project coordinator 1	Email (Newspaper)	August 2022
2	High school A	Teacher	Avatar school project coordinator 1	August 2022
3	Elementary school B	Teacher	Avatar school project coordinator 1	November 2022
4	High school C	Teacher	Avatar school project coordinator 1	November 2022
5		Teaching assistant	From No. 4	
6	Elementary school D	Teacher	Avatar school project coordinator 1	November 2022
7	No Isolation Germany	Staff	Email	November 2022
8	Hospital 2	Avatar school project coordinator 2	A student's parent who was introduced to us by No Isolation	December 2022

#### 2.3.2 Recruitment and study participants in Japan

Initially, an interview was conducted with a representative from OryLab Inc. in October 2022. Subsequently, through their referrals, we were introduced to representatives from prefectural^1^ boards of education (BOE) and teachers from schools implementing OriHime. Using snowball sampling, at the end of all interviews, we asked representatives from BOE or schools to introduce us to teachers and students using OriHime in the classroom. We were introduced to teachers from high schools and special needs schools, and in the case of one special needs school, also to students. However, since the Japanese government only downgraded COVID-19 from a pandemic to a regular infectious disease in May 2023, we were not granted access to schools for participant observation. As a result, we conducted interviews in Japan from January to March 2023 and did so with: staff members from two prefectural BOE, teachers from two special needs schools and one high school, and students from a special needs school who had used OriHime. In some cases, as shown in Table 3, multiple individuals participated in a single interview, resulting in a total of 14 individuals being interviewed. For this article we did not include the individual student's experiences. Therefore, this article is based on a total of eleven interviews from the Japanese side.

**Table 3:** Interview participants Japan: information on affiliation, recruitment route and interview setting.

No	Affiliation	Role	Recruitment route	Date interviewed
1	OryLab. Inc	Staff	Email	October 2022
2	Education board prefecture E	Staff	From OryLab Inc.	January 2023
3	Special needs school F, a hospital school	Vice-Principal	From OryLab Inc.	February 2023
4		Teacher 1		
5		Teacher 2		
6	Special needs school G	Principal	From OryLab Inc.	February 2023
7	Special needs school G	Teacher 1	From No. 6	March 2023
8		Teacher 2		
9	Education boards prefecture H	Staff 1	From OryLab Inc.	March 2023
10		Staff 2		
11	Technical high school prefecture H	Teacher	From No. 9	March 2023

### 2.4 Data collection and analysis

We prepared interview guides for all groups of study participants that included open-ended questions. Since we refer in this article to study participants with experiences in implementing avatar robots at schools, we present example items of our interview guides for coordinators and educators in Table 4.

**Table 4:** Interview guide.

Example questions to coordinators and educators
- How did the idea of using avatars at schools come about and what happened leading up to implementation? - Who is involved in the project and responsible for what? - How is the project funded? - What are/were the challenges? - What is the relationship with the avatar’s developer? - What impact did the COVID-19 pandemic have on the use of avatars?
- Who is eligible to use an avatar and who is not? How is access granted? - What have been the experiences using avatars in the classroom/school environment? - What are the challenges?
- What are the experiences interacting with students through the avatar? - How do the classmates react and interact with the avatar? - If you could redesign the avatar yourself, what would you change (add or remove, design differently)? - Have you used the avatar yourself or could you imagine using it? - What do you think about the use of avatars in the future? What impact do avatars have on society?

Given that the first author speaks German, she conducted the interviews in Germany. All interviews were planned to be in person. However, due to a COVID-19 infection, most interviews were conducted via Zoom instead. The interviews lasted between 40 and 100 min. Face-to-face interviews were conducted at schools or participants' workplaces; participants in online interviews were at their homes, or their workplaces.

In Japan, the two authors and a student research assistant conducted online interviews in Japanese via Zoom. Since the second author is a Japanese native speaker, she facilitated the interviews. The second author and the student assistant asked additional questions. The interviews took place at the participants' workplaces. Each interview lasted approximately one hour due to strict schedules of BOE members and teachers.

All participants gave informed consent to take part in the interviews and for the interviews to be recorded. The interviews were transcribed verbatim and the German interviews translated to English for internal use.^2^ The first author formulated the research questions for this article and discussed them with the second author. The research questions then served to guide the analysis and both authors searched through the interviews, gathering the relevant information for each single case in both countries on the (1) process of implementation, (2) areas of application and eligibility and (3) potential and limitations of avatar technologies from the viewpoint of our study participants. The authors first discussed the findings for both countries separately. We compared and contrasted different cases within the country using paper and pencil methods. The information on structural aspects was also compared with the information from the media search to assess whether there were any indications of the prevalence of these structures. We then compared the two countries to identify structural similarities and differences.

## 3 Findings

In accordance with our three research questions for this article, we group our findings in the following sections: In 3.1 (Germany) and 3.2 (Japan) “Setting-up avatar school projects and implementing the avatar at schools,” we detail who is involved in setting-up avatar projects at schools in each country, who funds the avatars, how the implementation processes is carried out, how regulations and legal requirements are handled, and what organizational or technical obstacles the implementing bodies face. Since the implementation process differs in Germany and Japan, we provide our findings separately for both countries. In 3.3 we present the “Areas of application in schools, access and eligibility to avatar technologies,” for both countries together and highlight similarities and differences. In 3.4 we focus on “Potential and limitations seen by our study participants” regarding avatar technologies in schools in comparison with conventional solutions (such as hospital schools, home tuition or sending schoolwork to the child) on the one hand, and video conferencing tools on the other hand.

### 3.1 Setting-up avatar school projects in Germany and implementing the avatar at schools

According to a representative from No Isolation Germany, AV1 has been used in German schools since 2018, and by November 2022, there were 279 AV1s in German classrooms, from elementary school to high school. AV1 can be rented from No Isolation, but in most cases, it is bought for € 3,900 and comes with a package including insurance and Wi-Fi plan for € 90 a month or € 860 a year. Although there are cases where parents or schools organize an AV1 for a single child, in most cases non-profit organizations (NPOs) are involved in funding the avatar through donations. There are single projects where a health insurer was involved in providing AV1 for a limited period. However, attempts from parents to have health insurers cover avatar costs as a regular aid were denied. City or federal state school boards sometimes support avatar projects, or media centers distribute the robot. The longest-lasting projects are cooperations between NPOs that fund the avatar and major hospitals in cities such as Munich (23), Berlin (24), Düsseldorf (25), and Hamburg (26). Such hospitals select participants and distribute, implement and maintain the avatars.

The avatar school project at coordinator 1's hospital was one of the first avatar school projects in Germany. The implementation was bottom-up and the coordinator of this project remembered how they answered the request of individual patients to stay connected with their classmates and keep up with the curriculum:

The whole thing took off […] around 2014. A patient, 16 years old, a high school student, made a request. […] I then tried to implement it with him as an individual case. We worked with a laptop with two microphones, set it up in the classroom and wired it up. Tried out the software. Looked at how it could be done. This worked as an individual case, but it was incredibly time-consuming. […] It worked with these means, but it didn't work well, let's put it that way. But the patient used it anyway and our internal evaluation showed that it gave him a lot of pleasure and also a bit of confidence that an external body had taken care of the issue “what about school?” And then we continued and tried out various other systems. (Germany, coordinator 1)

The hospital team evaluated its trials and developed own criteria for a good system: Most important was good audio quality (even more important than video quality,) followed by the maximum possible autonomy for the child, and the system should be as simple as possible for teachers to use. Coordinator 1 finally came across AV1 in 2018. AV1 fulfilled all three criteria and the hospital team was satisfied. They cooperated with an NPO to fund the first avatars through donations. After two successful years, the federal state's school board joined the project and funded additional AV1s.

Generally, the avatar is not meant to be a long-term solution. The aim is that the student returns to school and the avatar is passed on to another child. To grant equal access to education, the avatar is provided by a coordinating body that distributes and maintains it. According to No Isolation, AV1 is not intended to be a product only for well-off individuals or families. Therefore, the manufacturer searches for partners such as NPOs, media centers, local politicians, school boards or healthcare insurers to fund the avatar and make it accessible to the general population. In another hospital, AV1 devices have also been bought with donations and the annual service packages have been funded by an NPO. The hospital lends devices free of charge to the schools where they are used. Both coordinators see cooperation between a funding body and a hospital as essential for a sustainable usage and setting up the basic infrastructures. The coordinators' role is to select and inform the children and their parents and provide informational material. The way and degree the coordinators engage with the schools, however, varies.

Coordinator 2 mainly acts as mediator and provides information. Coordinator 1 visits the child's school to give a lesson on technical aspects of AV1 and inform their classmates about the disease and how illness can lead to social isolation, and the importance of social and educational participation. The student joins the class through the avatar for the first time and together they select a desk where the avatar is placed. In many cases a place in the middle is preferred. Here, the student can see the board and have the feeling of being in the middle of classroom activities. Both coordinators also provide technical and psychosocial support and communicate with No Isolation in case of hardware problems. They agree that providing information is crucial for a smooth implementation. The workload of schools and teachers and parental engagement seemingly affect the implementation. Both coordinators experienced slow and frustrating processes at schools where principals and teachers had a high workload and/or were not enthusiastic about the avatar:

There are always cases where the school takes a terribly long time to go through this approval structure. Where everything is delayed. In the end, we sometimes can't provide [an avatar] for a patient because it really, you'd be amazed, takes months for something to happen. And then you can't get hold of the school principal, and he passes it on and that's where it somehow often gets stuck. Then parents [of the classmates] have questions. Then a parent initially says: “No, I don’t want that. Because Big Data and America are monitoring.” (Germany, coordinator 1)

In contrast, implementation went smoothly at schools with higher capacities for using emerging technologies, teachers who understood the needs of students who are ill and were enthusiastic about the avatar, and where parents were fully on board. In most cases, the avatar was being used in the classroom for the first time, so structures had to be established. When a second avatar was used at the same school, coordinator 1 experienced they could rely on established structures and preparations proceeded faster.

The biggest issue implementing the avatar at schools is how the European General Data Protection Regulation (GDPR) is interpreted. Before the avatar can be implemented at school, its usage must be approved and regulated by the federal state. Whereas, according to No Isolation, in some federal states the approval went quickly and smoothly, in others there were many issues which needed solving. The usage—and accordingly the settings of AV1—is limited to one person and password protected. Photographing and video recording is not possible and not allowed. Furthermore, Germany follows an opt-in option, which means that in each class every single teacher and child or their guardians must give their written consent. Both coordinators have experienced that the school implementation failed because the school, individual teachers or other students or their guardians did not consent. If an individual teacher does not consent, there is still the possibility of not attending this teacher's lessons. If one of the classmates does not consent this means that the avatar cannot be used at all. One German coordinator explained:

We ourselves consider the avatar as very high level in terms of data protection, meaning with very high standards. However, there are still reservations. […] And if there is someone who does not agree, then it cannot take place. This also has the disadvantage that it is a higher entry hurdle […]. I have a case right now where it's on the tipping point. I can't tell you how it will turn out. (Germany, coordinator 2)

One of the concerns teachers often mentioned was that parents could watch their lessons through the avatar. Therefore, the child must wear a headset and parents have to promise they will not overhear the lessons. As a teacher from an elementary school recalled, establishing a trust-filled relation with the parents eased her concerns:

Well, at first, I was unsure because I didn't know what it would be like in the family, at home. Can they follow all my lessons and know what I’m saying and doing? […] [Y]ou're insecure when you feel like you're being watched and can't really assess how he's [the student] reacting because I don't have any feedback in terms of facial expressions or anything. His mom actually took this insecurity away from me. […] I was there and brought materials. Then she said: “It’s so cute when he sits in the living room in front of his laptop and talks about something. And I don’t hear anything.” And then I knew that the only communication was between him and the class. […] This took away a bit of my fear. (Germany, teacher elementary school B)

Teachers also discussed technical concerns. It was important that the operation be simple. The fact that AV1 only has one button to turn it on meets this requirement. Another hurdle can be the internet connection, either at school or in the student's home/hospital room. Bad audio quality can also obstruct participation even more so than poor video quality.

### 3.2 Setting-up avatar school projects in Japan and implementing the avatar at schools

The implementation of OriHime at schools was initiated in 2015 by OryLab. Inc in collaboration between a foundation promoting education and a special needs school.^3^ A teacher of a special needs school explained that hospitalized children were entitled to use OriHime to visit places of their choice and their former schools via OriHime before their return to school to make the transition smoother. He further stated:

Even though they [the students] are returning to their former schools, if they go back suddenly and if they meet suddenly with friends they haven't seen for a long time, they are very anxious about going back to their former school. So, we started using the OriHime robot to communicate with the students on the other side on a regular basis to help the students over there get used to it, and also to help the hospitalized student get used to the idea of going back to the former school. (Japan, teacher 1 special needs school F)

In another prefecture a similar pilot project was set-up between the prefectural BOE and an educational foundation in 2017 and expanded in 2019 to all prefectural schools after successes in educational participation and smooth returns to school.

According to OryLab, Inc., there is no available data on the total number of devices used in Japanese schools. At the time of our interviews, OriHime could not be purchased^4^ and renting was the only option for its use in schools. The entities renting OriHime for school usage are the BOE or individual schools. There are two main methods of funding the avatar: participating in projects sponsored by foundations promoting education or from the prefectural/school budget. These methods are not independent but interrelated: OriHime is often first introduced through pilot projects supported by foundations in cooperation with schools (e.g., special needs school F) or the prefecture BOE (e.g., prefecture E). If its effectiveness is recognized, it may be continued with the prefecture's own budget. In other cases, the school (e.g., special needs school G) or prefecture (e.g., prefecture H) secures a budget from the beginning without support from a foundation. Renting OriHime requires annual renewal and the number of OriHime rentals each year may vary due to budget constraints. Both prefecture E and H have implemented systems that allow OriHime rental for any school within both prefectures. In the case of prefectural schools, the schools apply directly to the prefectural BOE. However, for schools under jurisdiction of municipalities, they first submit their applications to the local government, which then transfers the applications to the prefectural BOE. This process can be complex and time-consuming. According to a study participant, the prefectural BOE should lend the start-up funds and if the effectiveness of OriHime is confirmed, he suggested that municipalities secure budgets and directly lend OriHime to schools within their jurisdiction, thus eliminating the impact of budget fluctuations.

However, it is difficult for some municipalities and individual schools to obtain budgets. According to news reports, renting an OriHime robot, microphone and receiving equipment for three-months costs JPY 136 000 (27).^5^ Special needs school G managed to introduce OriHime and rent it using a special budget allocated for the school's events. Funding it through a permanent budget remains challenging, though. The school might be able to acquire additional subsidies to rent OriHime for a few months, but there are limitations to its continued operation.

Participants described various implementation barriers involving a school or hospital accepting OriHime or not, and whether an organizational structure made implementation feasible. For example, special needs school F operates a hospital school and engages in implementing OriHime at student's former school to support return to these schools. Special needs school F must first explain the issue of returning to school and why OriHime might be helpful to various departments—and make sure this information has been understood correctly. This process can be lengthy and sometimes a hospitalized child has been allowed to go back to school before receiving permission to use OriHime there. The schools must ask the district BOE for permission and some districts do not allow the implementation due to personal information or privacy concerns. According to special needs school F, the main hurdles implementing OriHime at schools have been a lack of awareness for the needs of students with an illness and not having precedents and regulations in place for these new technologies. As the vice-principal stated:

[W]hen it comes to using OriHime, they will not accept something that has never been done before. They would say, “Can we allow that?” The response from schools and school boards is the same, “No, no one has done it before, so it’s not allowed.” […] Anyway, the biggest issue is the awareness of education for [students who are] sick and weak and the recognition of OriHime. I think the most important thing is to improve this. (Japan, vice-principal special needs school F)

Since there are no regulations regarding implementation or privacy, the implementing teachers or schools have established their own rules concerning privacy protection. They drafted informational materials to use for informing teachers and parents and raising awareness about the conditions and needs of students who are sick. They usually write a letter to the parents of the children in the class to explain and ask for permission, although this is not a legal requirement. In case OriHime is used from the hospital room, the schools for children with special needs also require the hospital's approval. They constantly exchange information with the physicians and nurses in charge, as well as the children's guardians, while also collaborating with psychologists and medical social workers.

When OriHime was to be operated from home, our study participants also reported parental concerns about video conferencing tools for schooling opening a window into their home environment. When the parents learned that OriHime only streams the classroom in one direction they were reassured: “When students take classes using OriHime from home, the parents […] are very relieved to know that the school cannot see what is going on at home.” (Japan, BOE prefecture E)

The implementation process was smoother at schools that already had experiences with OriHime. However, how smooth such implementations went not only depended on the individual teacher's awareness, but on administrative processes. For example, if a teacher in favor of and experienced with OriHime should be transferred, the implementation of the avatar at the new school might be subject to other complications.

OriHime comes with an iPad, is equipped with its own Wi-Fi and can also use local Wi-Fi, enabling students from households without Wi-Fi or lacking a mobile device to remotely control OriHime. However, participants shared that issues have arisen in schools with poor network environments, causing frequent disruptions in communication. Parents then had to contact the school and technical staff and/or teachers had to visit the classroom for troubleshooting. While many study participants mentioned that OriHime itself was easier to set up compared to Zoom, using OriHime entailed additional tasks for educators, such as charging the device and turning it on in the morning, adding to their regular duties. In some cases, a device was stationed at the school for the rental period. Yet in one case, teachers from a special needs school had to pick up OriHime and bring it back to the prefectural BOE each day it was to be used.

### 3.3 Areas of application, access and eligibility to avatar technologies in Germany and Japan

We identified three areas of avatar application among our cases from Germany and Japan (Table 5), which differ in terms of the child's condition and school type where the avatar is used and accordingly follow distinguished purposes of use. Type one “hospitalized children or children recovering at home who use the avatar at (a) hospital schools and/or (b) their former school” can be found in both countries, whereas type two “children with physical, mental or developmental disabilities at special needs schools”, and type three “children who refuse to go to school (despite good relationships) or feel pressure/sick entering school” were only present in Japan. In the following section, we present all three areas of application and further elaborate on the purpose and duration of use, and who is eligible for operating an avatar at school.

**Table 5:** Areas of avatar application.

Student condition and school characteristics	Earning academic credit	Maintaining relationships	Smooth return to school	Internship & work experience	Motivation to go (back) to school	Country	Duration of use
1 Hospitalized children or children recovering at home who use the avatar at (a) hospital schools and/or (b) their former school	✓ (only 1b)	✓	✓			Germany and Japan	A few weeks to a few years
2 Children with physical, mental or developmental disabilities at special needs schools		✓		✓		Japan	Irregular event basis
3 Children who refuse to go to school (despite good relationships) or feel pressure/sick entering school	✓	✓			✓	Japan	Average a few weeks to months

#### 3.3.1 Hospitalized children or children recovering at home who use the avatar at a hospital school and/or at their former school

In both countries the avatars are used for students with childhood illnesses, students undergoing treatment or who must isolate to prevent infections and cannot attend school in person for a longer period. They either use the avatar from the hospital or from their home. In Japan, when a special needs school operates a hospital school at the hospital the student is hospitalized in, the responsibility for this student is transferred to the special needs school. In these cases, the special needs schools oversee implementing OriHime (as in our cases from prefectures E and F). If the hospitalized student has to isolate, the teachers utilize OriHime in the hospital school so that the student can participate from the hospital room. Furthermore, the teachers use OriHime to assist students to return to their former school. OriHime is brought to the former schools at least once a month, with the frequency increasing as the time of return approaches. There are also cases in Japan, for example in prefecture H, where no special needs school is involved and high schools and their students apply directly at the BOE to use OriHime.

There are also cases in Germany where AV1 is provided by a hospital school and used, in addition to the hospital school classes, in the former school (23, 25). However, when there is no hospital school, students are eligible for several hours of private lessons a week during hospitalization and the recovery phase at home. Many students from our cases used this conventional system in addition to attending lessons via the avatar at their original school. AV1 is distributed by the psychosocial hospital teams and eligibility decided by the physician. Since in Germany the avatars are often funded through donations for a certain disease, the purpose of the donations is oftentimes also linked to patients with that disease. At one hospital, the project was expanded to include children from another ward after the federal state's schoolboard joined the project. Further extensions are planned to gradually provide access for all children in need. However, the initiatives start where structures are already in place. This means patients without a lobby come last.

Furthermore, one coordinator from Germany stated that access to education and social participation is unequally distributed. Children whose parents have a high commitment and care about their child's education have a better starting position in the education system. This is also true for the use of avatar systems. The higher the hurdles for the implementation of the avatar, the more engagement from parents is needed for children to use the avatar at school. High data protection requirements can thus have the effect of reinforcing unequal access to the education system:

When it comes to participation, it’s always about justice. And here, too, we notice that it is unequally distributed. Because some families are incredibly well-connected, they are simply an advocate for their child. Often, they can create better starting positions for their child. And I believe that the avatar as it is currently positioned, also further exaggerates this. This high level of data protection and the like, this high threshold that has to be crossed first. (Germany, coordinator 2)

In contrast, in Japan the BOE and schools oversee distribution and decisions on eligibility. OriHime is funded by budgets from the prefecture or school, and not limited to certain diseases or conditions. Educational authorities determine the duration of usage and this varies. For example, prefecture H limits usage to one month, while prefecture E permits two months to meet the needs of several students. There is the possibility of extension. In Germany the duration of use corresponds with the period the student is unable to attend lessons in person. In case of coordinator 1's hospital, two thirds use the avatar from home, while the other third uses it during hospitalization. The users range from elementary to high school students. The usages vary between several weeks (although in terms of appropriate effort a longer period is intended) up to several years. In both countries, if there is no avatar available, the child's name goes on a waiting list. It might happen that a child is able to go back to school in person before a device becomes available. This difference in distribution between Germany and Japan is also reflected in how access to the device is handled: In Germany, in compliance with data protection regulations, the login to AV1 is personalized for the period of usage. Only one student per usage period can create an account for one device and is allowed to use the login data. In Japan, the login to OriHime is password protected, but not personalized. That means, if the teachers hold the login data, they can use it for several students during the same period.

In both countries the purpose for implementing avatars is social and educational participation and a smooth return to school. In Japan, a member of prefecture E's BOE referred to a lack of opportunities for learning and making experiences, which might result in limitations of physical and mental development and social participation of hospitalized students, to explain the purpose of using OriHime at schools:

The background of this project is that students at special needs schools, elementary schools with in-hospital classes, and those who are undergoing medical treatment for illnesses or physical conditions that make it difficult for them to go outside, have difficulties participating in learning and lack opportunities to gain experiences. The prefectural government has been aware of the fact that this is a serious limitation for their mental and physical development, and their participation in society. (Japan, BOE prefecture E)

In Germany, coordinator 1 explained that the everyday life of children with a long-term illness is often dominated by the disease. Therefore, the aim is helping such children stay part of or reintegrate into the social school routine, maintain their social contacts, and regain a piece of normality and a regular daily routine beyond the disease: “We want reintegration into everyday school life. To convey the feeling of being involved. If possible, the patient should be given a bit of a sense of normality. It's not so much about academic achievement and lesson content.” (Germany, coordinator 1).

Although maintaining social relationships is central, in both countries keeping up with the curriculum and earning credits is also important, especially for older students. For example, some of the high school students in Germany have taken their exams via the avatar. Coordinator 1 reported the case of a student who took her exam in the hospital room under supervision by a teacher, while the avatar was among the other students taking the exam at school at the same time. In Japan, the Ministry of Education, Culture, Sports, Science and Technology (MEXT) issued guidelines during the COVID-19 pandemic on the conditions for remote learning (28). The MEXT guidelines allowed students to receive academic credit for attending classes online without physically attending school. Prior to this, for students unable to attend school in person, learning support using video conferencing systems or OriHime was possible, but such attendance was not officially recognized and academic credit could not be obtained. By utilizing OriHime to attend classes and receive academic credit, some students who had been using this technology in schools for extended periods (e.g., high schools in prefecture H), were able to benefit from this recognition.

Besides attending lessons, avatars are used for informal talking with classmates and friends during breaks. In both countries avatars can be taken on school trips or excursions and are used to join cultural events. One German teacher recalled how her student used the avatar for the first time to participate in a school trip:

It was good timing because we were going on a school trip in February. And he [the student] was sad that he couldn’t go. But the avatar was able to go. […] We, for example, carried it in a rucksack, with the head peeking out. On a night hike, it lit up like that. That was really great. I don't know if he saw many of the stars and such, but he was there somehow. […] And I think that was very important for him. (Germany, teacher high school A)

Despite these described ways for students to use the avatar in German and Japanese schools, participants in both countries also shared instances of children not wanting to use the avatar. Reasons participants cited for this refusal included children feeling too shy, or thinking they were not good at robots. Other children did not want extra efforts made that put them at the center of attention. Teacher 1 at special needs school F explained:

Some children are very nervous when they are using OriHime, for example, they cannot even reply when someone calls out to them. I realized how difficult it is for children who are sick to communicate with their former schools, even using OriHime. So, I think that's one of the reasons why they are turning it down. They don't want to use it, or they don't have the confidence to use it. (Japan, teacher 1 special needs school F)

Furthermore, some students who had not been well-integrated into their class before having to be absent said they were not interested in using the avatar to stay socially connected. As our participant teachers reported, good integration into the class and a well-functioning class community are advantageous when using the avatar. Our German elementary school teachers pointed out that committed parents and a good relationship between teacher and parents are also important in case of younger students. In contrast, coordinator 1 reported that a student with poor integration was bullied with the avatar: Other students obstructed its view or turned it to the wall when the teacher was not looking.

#### 3.3.2 Children with physical, mental or developmental disabilities at special needs schools

In Japan, OriHime is also used for children with disabilities at schools for children with special needs. In contrast to hospitalized children, the usage is not on a temporary basis, but long term and on a regular basis. For example, prefecture H introduced OriHime for students with severe disabilities who receive home schooling to integrate them into a class at a school for children with special needs. Sometimes their teachers also take OriHime for a walk in the neighborhood or at the school to show the student around. OriHime is also used regularly for students with developmental disabilities, for example for students with sensory sensitivities for whom noise is challenging. A BOE member reported:

For example, when students with sensory sensitivities have difficulties participating in group classes, such as music classes, they are able to participate via OriHime from a separate, quiet location, even though they are still in the same school. This is a regular use of OriHime for a couple of hours a week, for example. In addition to this, OriHime can be used for events, exchange activities with other schools or the local community, and so on. This is more of a one-time use, on an irregular basis. The current situation at special needs schools is a mix of such regular use and one-time use. (Japan, BOE staff 2 prefecture H)

On an irregular basis OriHime is also used for participating in events, exchange activities with other schools or the local community, or visiting the neighboring classrooms, with one classroom operating OriHime and the other watching it. Furthermore, since classes in schools for children with special needs are small, OriHime is used to connect students from the same age group across schools.

Whereas in the above-mentioned cases the purpose is to connect the students to the school social environment and with peers, there are also cases which go further. These aim at connecting students with disabilities to society and offer alternative work opportunities. There have been several attempts to use OriHime for work experience for students, including programs offered by the OryLab Inc., in which students from special needs schools in several cities remotely served customers in cafes.^6^ Special needs school G set-up their own internship program for students with severe disabilities to play an active role in society post-graduation. The program's aim is that students gain first work experiences and interact with society. A teacher from this school explained:

One of the major initiatives we are considering is the use of OriHime for practical training outside the school, which was also conducted this school year. We are thinking of taking OriHime to places where students actually go out and work or interact with society and give them some kind of practical training experience for a set period of time, from one to three days. […] One of the students [from this year], […] has a severe disability, and it is difficult for him to go out and work because he is not able to move his body. However, with the use of OriHime, even with limited physical movement, he can use a tablet to make OriHime perform actions or convert voice input to speech and enable him to talk to others. Using such tools can lead to simple work. (Japan, teacher 1 special needs school G)

The main challenge for the teachers is fostering students' ability to communicate, especially with people they do not know. The teacher responsible for the career guidance at this school plans to incorporate OriHime in the process of finding out where the students will go post-graduation, match them with the right places, and help them transition to a place where they can live after graduating. This type of usage goes beyond the school environment to connect students to society and is not considered short-term.

#### 3.3.3 Children who refuse to go to school or feel sick entering school

In the German cases the avatar use was limited to children with childhood illness or chronic conditions. However, both hospital coordinators reflected on the use of avatars outside of their program. Autism was seen as a condition that avatars could support well. In the case of anxiety disorders or depression, however, it should be carefully considered whether symptoms such as avoidance or social withdrawal are intensified by avatars and thus, their use might be contraindicated. One coordinator stated:

[T]here are many people who can’t go to school because of psychiatric illness. Well-justified reasons for not being able to go to school. And here, of course, it is important to think about which symptoms I am actually supporting negatively when I set up an avatar. So, if I have someone with an anxiety disorder or a depressive episode, am I pushing them even further into avoidance or withdrawal if I give them this opportunity? Because, actually, the goal is to go back to school. If we have a child with autism, there is no condition after autism. That means I would rather imagine that you could support someone in those moments when they can't go out and continue with the avatar. (Germany, coordinator 1)

In Japan, OriHime may be used for students who refuse to go to school in exceptional cases. School refusal is defined as being unwilling or unable to go to school due to psychological, emotional, physical or social conditions. Reasons for school refusal can vary and may be due to problems between students, such as bullying. In such cases, using OriHime to attend school may not be suitable. This is because OriHime is understood as a communication tool and students who do not want to communicate with their classmates or teachers have no desire to use OriHime. A BOE member declared that if these students want to attend classes, it is better to use other measures:

We have received many requests such as, “Can children who are avoiding school also use OriHime?” […] but the program is intended for children who are undergoing treatment for illnesses. For children who avoid school, our prefecture has a program against bullying and school refusal. This program called [name] is not an OriHime program, but a study support program using a tablet that can be used at home. (Japan, BOE prefecture E).

Although this is the official statement of the prefecture's BOE, the participant described a case where a student felt sick when entering the school. To ease the pressure of going into the classroom this student was allowed to use a separate room and log in to a spare OriHime in the classroom to see what was going on and sense the atmosphere. This helped the student overcome the pressure and eventually they were able to enter the classroom in person.

There are students, though, who refuse or avoid school despite having good relationships with their classmates and teachers. As one high school teacher reported, a student was positive about using OriHime to participate in classes but was unable to go to school for other reasons:

One of my classes had a child who had stopped attending school. I can't tell you why, but this child was in the first year [of high school], and we wanted to let him earn credits for the first year. […] We decided that OriHime was better than a camera, because OriHime could move its arms, clap its hands, and we could see what the other person was thinking and what was going on. (Japan, teacher high school H)

This child was allowed to use OriHime for a longer period and earn academic credits. The teacher stated in the interview that it was important to leave open the possibility that the student would be motivated to return to school in the new term. This was because the student was not attending school but wanted to go, and also due to the student's interactions with friends.

#### 3.3.4 Potential and limitations of avatar technologies

Regarding the potential of avatar technologies our study participants in both countries referred to the opportunities of social and educational participation. In most cases avatars did not replace conventional ways of securing education—such as hospital schools, home tuition, or sending school materials to the child—but were installed in combination and answer to the social needs of the students. According to the experiences of the coordinators from both German psychosocial hospital teams, avatars have potential to stabilize patients' psychosocial condition, motivate them, and thereby secondarily, support the medical treatment. A positive affect was that because the students remained in the school social environment, they maintained confidence and a perspective on how to move forward. This may have helped them cope with their illness. This was also mentioned as a benefit for school reintegration. Children who are absent from school for a longer time lose their connections and the hurdle of having the courage to go back to school and reintegrate into normal everyday routines remains high. As coordinator 2 pointed out, this directly connects to equality of educational opportunities and the child's future chances:

It's about participation, but of course in social life as well as in school. I think it can often be an advantage for our patients when it comes to coping with their illness, because you still have a perspective. You don’t have the feeling of being out of it […]. And in this way, you can simply minimize the acquisition of an additional illness or the persistence of an illness […]. So, there's the health aspect, and of course the social aspect. Because when you're at home a lot, it’s difficult to maintain contact with others, with people your own age. And that is a matter of educational equality. Because, as I said, it's about what kind of opportunities you create for them or they create for themselves for everything that comes later. (Germany, coordinator 2)

Compared to other telecommunication devices, such as Zoom, the avatar was described as representing the student in the classroom, providing a physical presence, an ability to act and autonomy in the classroom. A BOE member from Japan explained:

One of the great things about OriHime is that you can take action. […] For example, rather than just having a computer on a desk in the classroom, with a blackboard or whiteboard facing away from it, OriHime can be there, moving its head occasionally, or raising its hand […]. I think it is a unique feature of OriHime that the children around it can feel it. If there is only a computer, the children will not be aware that there is a child at the other end of the computer. […] But with OriHime, there is a sense of presence, and when they come back to school, it is not as if it has been a long time. (Japan, BOE prefecture E)

Through the possibility of gestures (OriHime) or facial expressions (AV1), the avatar can facilitate communication with its reactions, and it is as if the absent student is with their classmates all the time. Accordingly, when the student is allowed to go back to school, there is a feeling as if they had never been away. Therefore, the avatar can reduce fear and insecurity in interactions and counteract anxieties. The presence of the avatar also makes a difference in the awareness of the classmates. The anthropomorphic design was interpreted as supporting this representational function, fostering the connection and communication, and reducing hesitation on the part of the classmates who do not know what to talk about with the student who is sick. One coordinator from Germany reflected:

This idea of putting a figure there and having it represent the absent person in a certain way somehow works. I can't say exactly why it works. But it works. And if I put a monitor there, then the person is somehow there, but something is missing. I think there's a protective function missing for our patients. […] And perhaps it is also easier for the class community to interact with them when I put something in between, the interaction becomes more natural. […] [M]any people don’t know how to interact. There's someone who isn't allowed to go to school. They are isolated. They have a very serious illness. (Germany, coordinator 1)

Another aspect of this physical representation in the classroom is that the student who cannot attend in person does not disappear from their classmates' and teachers' awareness and is not forgotten. In addition, the one-way video transmission was also seen as an advantage for the students who are sick as this protects their privacy. Unlike other videoconferencing or telepresence technologies, where video can be turned off, there is no need to justify not turning the video on, as one BOE member from Japan discussed:

[T]here are children who do not want to be seen by others. Some children are undergoing treatment, or have difficulties in interpersonal relationships. Or, since the video is sent from home, parents often say that they don't want what is going on around the house to be seen. With online tools, if you stop the video, it turns off, but that gives the impression that you are shutting it off, but in the case of OriHime, you can't see the operating side by default. In that respect, I've heard that it is very easy to use. (Japan, BOE staff 2 prefecture H)

Moreover, our Japanese study participants reported cases where OriHime was introduced for children with disabilities to integrate students educated at home into a class community, provide transitional or alternative options of attending classes for students with developmental disabilities, foster exchange between schools or with the local community, or provide internship opportunities and thereby connecting students with society and future work opportunities. In addition, some Japanese schools tried to use OriHime for students who refused to go to school, but were interested in maintaining communication and relationships with teachers and classmates. Here, OriHime functioned as maintaining the connection and keeping up the motivation of coming back to school.

Study participants in both countries agreed that a successful usage depends on how the teachers integrate the avatars and create awareness for the students' situation among peers. The teacher's involvement and awareness of children's needs are crucial factors for how well the avatar and its user become integrated into the class and to ensure successful reintegration into the educational system. Despite the easy usage of both avatars, integrating them into the class community and raising awareness for the condition of the absent student adds to teachers’ workloads. In cases where learning materials are not readily available online, this also means additional preparation time and sending materials home or to the hospital. One German teacher summarized what is important from her point of view:

Good organization or coordination is necessary or is conducive. […] I think that keeping in touch with the parents in addition to the avatar is also important. And I also think that certain tools can support the whole thing, like the tablet, where the student can simply look up everything again. These are the pages that are being worked on in class. That's the homework again. […] Basically, of course, the child has to be interested in taking part and, yes, sitting there […] alone every day and turning on the avatar again and again requires, I think, a lot from the children. […] And for the parents it's also difficult because it requires a lot of support in my opinion. Apart from that the children have to have a certain tolerance, or the class has to show consideration. (Germany, teacher elementary school D)

Several other German participants also highlighted the importance of parental commitment and support and said staying in contact with parents was helpful. With high school students, personal communication or text messaging with peers was also referred to as an additional supportive factor. In contrast, our Japanese study participants rarely mentioned parental support and especially the teachers from special needs schools seemed to take over supportive roles.

Despite the avatars' potential our study participants from both countries also referred to several limitations. Although avatars can promote social participation, our German study participants stated that it can also have the opposite effect. As one German coordinator said:

I think if pupils already have a tendency to withdraw, are very shy, very avoidant, ashamed that so much extra effort is being made because of them, then it doesn't always make sense to use the avatar. In that case, it may be better, or it may be more sensible to try to actually bring them to school, if possible, and rather do less. So, I think you also have to make sure that you don't encourage withdrawal. (Germany, coordinator 2)

Also, the Japanese study participants mentioned that avatar technologies are not suitable for all students and it should be carefully considered in each case whether its use makes sense or if children need further support to feel confident using the avatar. Besides the physical constitution and illness, the willingness to communicate and interact are seen as prerequisites to a successful usage. This is also attributed to the character of the student or their social integration into the class community. Some hospitalized children are not confident enough to use an avatar for school. They are very nervous when using OriHime and cannot even reply when classmates call out to them. In addition, in Japanese special needs schools, where the avatar is used for children with disabilities, it might not be beneficial for children with severe intellectual disabilities who have difficulties understanding the concept of an avatar robot existing in a different place to them, as a BOE member stated:

When I was [still a teacher] at a special-needs school, I thought it would not be very effective if the students did not understand an alter ego was in the classroom at a distance. There are many students with intellectual disabilities in special needs schools, so it is honestly difficult for them to understand that an alter ego is at the end of the iPad. (Japan, BOE prefecture E)

Similarly, some study participants from Japan were of the opinion that younger elementary school students are less likely to benefit if they cannot understand the concept of an avatar robot. However, coordinator 1 and two elementary school teachers in Germany experienced the avatar as being successful with elementary school pupils, although younger children might need more support from parents and teachers.

When regarding the needs of children undergoing medical treatment, the one-way video transmission was understood as a protective factor. However, it was also seen as a disadvantage. For example, a German elementary school teacher reported that although seeing a child in the hospital could be overwhelming for the classmates and therefore no video is also a protective factor for the class, seeing the child from time to time or receiving a picture can help avoid shocked reaction on return to school in person. Furthermore, for teachers and classmates it takes a while to get used to this new form of communication. In the beginning it might be a strange feeling to know that video of one's self is streamed to another person they cannot see. Moreover, information communicated in physical co-presence such as body language and spontaneous or unconscious mimic is omitted. In addition, if students do not work with online material but paper and pencil, the teacher cannot see in real time what the student is working on and where help would be needed. For Japan it was mentioned in the context of exchanges between schools that the children enjoyed using OriHime for nonverbal communication with gestures, but also wanted to see each other's faces while communicating. Therefore, a special needs school in prefecture H uses both OriHime and Zoom at the same time.

## 4 Discussion

In the previous section we presented how AV1 was implemented at German schools and OriHime in Japanese ones. It is important to recognize that the presented cases allow an insight into the implementation processes in both countries and highlight tendencies, but since they represent individual initiatives and there is no single policy of introducing avatars at school, the generalizability of our results has limitations. In the following we will summarize our findings and contextualize them with the literature on telepresence robots and avatar technologies.

### 4.1 Implementing and gaining acceptance for avatar programs

Newhart et al. (3) pointed out that in the USA technological innovations at school have historically been introduced in a top-down process whereas telepresence robots are implemented in a bottom-up way. This was comparable to the initial initiatives in our German sample where psychosocial hospital teams answered requests from hospitalized students to stay socially and educationally connected. Yet, when the programs were recognized as successful and scaled up, one federal state's school board stepped in to support the program and made it available to children with other diseases. The service therefore became more sustainable and institutionalized. In our Japanese cases the implementation process was sometimes initiated by the developer in collaboration with foundations promoting education, the prefectural BOE or individual schools, or initiatives of prefectural BOEs and individual schools without collaborative support. Although in Japan the MEXT issued guidelines (28) for remote learning during the COVID-19 pandemic, neither in Japan nor Germany have avatar initiatives reached a policy level so far.

Comparing the German and Japanese cases, the most significant difference is that the initiatives in Germany are led by psychosocial hospital teams, whereas in Japan educational bodies take the initiative. One consequence of these different implementation processes is that in the German cases, coordinators from the hospital and/or parents have to inform and convince educators to accept and integrate the avatar in the classroom. As our participants explained, this can be a frustrating and exhausting task. This is also corroborated by a study from Norway (16). In contrast, in the Japanese sample the BOE or teachers from schools for children with special needs have to convince not only the schools, but also the hospitals to participate. As shown in the literature, implementing avatars at schools is a complex process, due to the intersection of education, technology and healthcare. As Newhart et al. (3) argued, developing a partnership between these sectors is a key factor for a successful inclusion of students via avatar technologies into the class community.

### 4.2 Granting equal access to avatar technologies

As our German cases show, cooperation between multiple participants involved—including parents—is also important for granting equal access to education. The implementation of avatar technologies at schools can mean that parents have to demonstrate high commitment and care about their child's education. Therefore, access to avatar technologies is not evenly distributed: Children with fewer educational opportunities due to their family background are more disadvantaged. Furthermore, unequal access to education can even be reinforced by high data protection standards when parents have to convince educators and other guardians to consent.

Conversely, in the Japanese cases, the commitment and high level of interest of teachers is important. If teachers have a busy schedule, no affinity to technology, or are not aware of the needs of children with illnesses, this might result in a reluctance to apply to the BOE for using OriHime in their class, thus preventing access to avatar technologies. Guidelines introduced by MEXT (28) allowing students to receive academic credit for attending classes online without physically attending school can be seen as an important step towards granting the right to education beyond physical presence.

Another issue is equal access to avatar technologies in terms of financial costs and program sustainability. In both samples funding bodies played an important role in implementing the avatars. However, in Germany the donations of the funding NPOs are often tied to a disease the organization targets. This therefore limits access to the avatar school projects. To guarantee a more equal access for all children, regardless of their condition, it is important that government institutions such as school boards, media centers, or health insurance companies get involved in funding and providing avatars. Whereas public funding through educational boards can be seen as broadening access to avatar technologies and granting a more independent use, the Japanese sample has shown how annual leasing contracts may be subject to budgetary constraints and how bureaucratic requirements can complicate the usage. Some of our Japanese participants suggested individual schools should purchase OriHime instead of renting, and thus eliminate the impact of budget fluctuations. However, the AV1 cases show that these kinds of avatar technologies often come with a service package, including Wi-Fi, insurance and maintenance fees. Therefore, it is necessary to establish mechanisms such as subsidies at the societal level to grant individual equal access to avatar technologies.

### 4.3 Protecting privacy

Privacy concerns was another issue that arose in both our samples regarding the aspect of the avatar bridging between the student's hospital room or home and their classroom. Implementing the avatar at schools involves negotiating understandings of privacy and data protection grounded in ethical values and legal norms.

In Germany the biggest issue implementing the avatar in the classroom is the European General Data Protection Regulation interpretation that each teacher, student and/or their guardians must give their written consent. Johannessen et al. (16) also referred to privacy as a key issue in their Norwegian sample, especially because the GDPR was enacted shortly before AV1 became available and uncertainties by the schools were high. Whereas in the Japanese sample there are no legal requirements regarding personal information for the implementation of avatar technologies at schools, this does not mean there are no privacy concerns from parents or teachers. On the contrary, the teachers implementing OriHime must explain and set up rules on their own carefully. A lack of precedent is often a high barrier to adoption because there is little incentive to introduce it.

Newhart and Olson illustrated how telepresence robots which transmit two-way video are a bridge between home and school and can bear a potential of violating the privacy of the home and of the classroom (6). Whereas the privacy of the home in our study was protected by the one-way video transmission of AV1 and OriHime, one major concern often mentioned by teachers in the German cases is that parents could watch their lessons through the avatar. This has also been reported in other studies, were teachers feared parents could comment on their classes on social media (6), or teachers being concerned that they lose control over access to their teaching when the classroom becomes extended by the avatar (16). Newhart and Olson (6) have proposed that school administrators should mediate between parents and teachers, clarify responsibilities and opportunities, set up rules for the use to protect privacy at home and in the classroom. This could be assured by a training session as a classroom aid for parents.

### 4.4 Enabling social and educational participation

The main aim of avatar robot school projects is enabling children who cannot attend school due to an illness, disability or extended hospitalization, to remain socially connected, regain some normality, keep up with educational requirements and avoid becoming socially isolated. In the German sample the avatar was clearly defined as a device developed for school—with special functions for the classroom. It should connect students with the school social environment and be restricted to a temporary use with the goal of smoothing the return to school. This was also the target and goal in studies from the literature (1–7, 9, 16) and for most projects in Japan.

However, in Japan the areas of application are broader. One reason can be seen in the differences in the implementation process. Since in the German cases the hospitals provide and manage the avatar programs with financial aid from NGOs supporting children with certain diseases, they define who is eligible and the area of application is tied to the needs of their patients. On the contrary, in the Japanese cases, where the avatar was stationed at the school for the rental period, teachers used temporarily unneeded devices and established new areas of application, exceeding the original idea of supporting students who are hospitalized or undergoing treatment.

One more reason for broader areas of application—the utilization for children with disabilities and internship initiatives—in Japan, is connected to the engagement of the developer OryLab Inc., which has opened a café in Tokyo where people with disabilities remotely work and serve the guest via OriHime (29, 30). Especially the internship initiatives go beyond school and aim at connecting students with disabilities with society and broadening their work opportunities. In this context, the avatar is not only configured as a device for temporary use, but as part of a new lifestyle that fosters a diverse and inclusive society. This is supported by the more universal design of OriHime that includes options for gaze-control. Moreover, this is part of the visions and policies of the Japanese government and science and technology development strategies to create the so-called Society 5.0 (31, 32).

### 4.5 Advantages of avatars depend on individual conditions and context

Findings from both countries indicate that avatars are not beneficial for everyone or in every context, and it is important to determine the advantages and disadvantages. In the German cases the avatars were implemented for children who could not attend school in person due to childhood illnesses. Autism was seen as a condition in which avatars could be supportive. However, in the case of anxiety disorders or depression, the psychosocial teams saw a risk that the avatar could intensify symptoms such as avoidance or social withdrawal and was thus not beneficial. Weibel et al. reported in an explorative study that the expectations of children with anxiety differ from those of children with cancer and neuromuscular diseases and found that: Children with anxiety expect to develop new friendships and social skills, re-enter the school environment with the avatar and eventually return physically, whereas children with cancer and neuromuscular diseases expect to stay connected with their peers and have the feeling of being present in the school and among them (2).

The Japanese cases showed that teachers had good experiences with children with severe physical and developmental disabilities. However, there were only individual experiences with children who refused to go to school. In both countries the participants stressed that the usage is only beneficial for children who have a good relationship with classmates and teachers and want to stay connected. In the German sample and in the literature (3) there are also examples of bullying through the avatar, which suggest that teachers should be aware and take care that absent students, who are already more vulnerable due to their condition, are not harmed.

Additionally, it is essential that students with mental disabilities or who are very young understand the concept of the avatar as an alter ego that represents them in the classroom. Furthermore, the literature (3, 6, 16) and our cases reported incidences of students who did not like being at the center of attention through the avatar. Johannessen et al. (16) also reported that children might reject using the avatar because seeing what they are physically missing makes being spatially disconnected even harder.

## 5 Conclusion

Our findings suggest that avatar technologies bear a high potential for children to stay socially and educationally connected. However, it is crucial that structures are established that grant equal access to avatar technologies and education. This means regulations by educational boards, recognizing academic credits for attending classes online or via avatar technologies, providing budgets for funding avatar technologies to make them accessible to the public without bureaucratic barriers, and establishing procedures and rules to protect the privacy of all involved without making the hurdles for implementation too high. Furthermore, guidelines or training on technical, educational and psychosocial aspects of avatar technologies for teachers are crucial for overcoming uncertainties, preventing harm and making the implementation a successful experience for all involved. Since our Japanese cases suggests that expanding the usage is promising, further research on the benefits for different student groups is needed.

## Data Availability

The datasets presented in this article are not readily available because the participants of this study did not give written consent for their data to be shared publicly. Therefore, due to privacy issues and the sensitive nature of the research, supporting data is not available. Participants disclose very personal issues, such as illness experiences. Although pseudonyms were used to protect the participant's identity, there is still a possibility that the potentially sensitive information could be identified by the details of experiences. Moreover, the study consent form and data sharing policy for this study as approved by the research ethics committee states that pseudonymized data may be used in scientific journals but just in cuttings/quotes and in a form not allowing to conclude the interviewed person. Since full transcripts cannot be fully anonymized, they cannot be shared with anybody outside the research team. Requests to access the datasets should be directed to Celia Spoden, spoden@dijtokyo.org.

## Footnotes

^1^Japan is administratively divided into 47 prefectures.

^2^Note: Interview quotes presented in this article have been translated from German and Japanese to English by the authors.

^3^Special needs schools often operate hospital schools. If students are hospitalized for longer periods and the hospital has a school, the student is enrolled with the special needs school.

^4^By the end of 2023, OryLab Inc. had released a new version of OriHime and offered it for sale.

^5^136 000 JPY is about € 871.34 (100 JPY=€ 0.64 in July 2023).

^6^An interview with a high school student with disabilities about her experiences remotely serving customers can be found here (in Japanese only): https://www.uhb.jp/news/single.html?id=33801&page=3
